# Local variations in the timing of RSV epidemics

**DOI:** 10.1186/s12879-016-2004-2

**Published:** 2016-11-11

**Authors:** Douglas B. Noveroske, Joshua L. Warren, Virginia E. Pitzer, Daniel M. Weinberger

**Affiliations:** 1 Department of Epidemiology of Microbial Diseases, Yale School of Public Health, PO Box 208034, New Haven, CT 06520-8034 USA; 2 Department of Biostatistics, Yale School of Public Health, New Haven, CT USA

**Keywords:** RSV, Epidemic timing, Prophylaxis, Harmonic regression

## Abstract

**Background:**

Respiratory syncytial virus (RSV) is a primary cause of hospitalizations in children worldwide. The timing of seasonal RSV epidemics needs to be known in order to administer prophylaxis to high-risk infants at the appropriate time.

**Methods:**

We used data from the Connecticut State Inpatient Database to identify RSV hospitalizations based on ICD-9 diagnostic codes. Harmonic regression analyses were used to evaluate RSV epidemic timing at the county level and ZIP code levels. Linear regression was used to investigate associations between the socioeconomic status of a locality and RSV epidemic timing.

**Results:**

9,740 hospitalizations coded as RSV occurred among children less than 2 years old between July 1, 1997 and June 30, 2013. The earliest ZIP code had a seasonal RSV epidemic that peaked, on average, 4.64 weeks earlier than the latest ZIP code. Earlier epidemic timing was significantly associated with demographic characteristics (higher population density and larger fraction of the population that was black).

**Conclusions:**

Seasonal RSV epidemics in Connecticut occurred earlier in areas that were more urban (higher population density and larger fraction of the population that was). These findings could be used to better time the administration of prophylaxis to high-risk infants.

**Electronic supplementary material:**

The online version of this article (doi:10.1186/s12879-016-2004-2) contains supplementary material, which is available to authorized users.

## Background

Respiratory syncytial virus (RSV) is a primary cause of hospitalizations in children worldwide [1]. Most children will have an RSV infection before 2 years of age [2]; however, certain high-risk groups are at an increased risk of severe RSV infection [3]. Prophylaxis (Palivizumab) can be given to high-risk groups including infants less than 12 months old at the beginning of RSV season who were born premature (earlier than 29 weeks gestation), infants with some congenital pulmonary and heart diseases, and immunocompromised infants [4]. Palivizumab has been available since 1998, and has been shown to be moderately effective in lowering the risk of RSV hospitalization [5, 6]. Each dose of Palivizumab protects for approximately 5 weeks; up to five doses are given to provide protection for high-risk infants, and the timing of these doses needs to coincide with the RSV season. Starting prophylaxis too soon is an inefficient use of resources, as the cost of Palivizumab is high and the risk of infection is low before the seasonal RSV epidemic begins; starting prophylaxis too late can put susceptible infants at risk for a severe RSV infection as they would not be protected when local RSV transmission begins. To optimize the use of resources and maximize the protection afforded to high-risk infants, it is critical to understand variations in RSV epidemic timing between locations so that prophylaxis can be appropriately timed [7].

Seasonal RSV epidemics in the United States can begin anywhere from early-autumn to late-January and can also differ in duration [8, 9]. The timing of the epidemics varies by as much as 10 weeks across the United States, with earlier peaks in the southeastern part of the country and later peaks in the north and west; on a more regional scale, earlier epidemics have been noted in urban counties compared with rural counties [10]. It is not clear, however, how these broad national and regional patterns of epidemic timing translate to the local scale. This study aimed to evaluate the timing and magnitude of seasonal RSV epidemics at the ZIP-code level in the state of Connecticut and to investigate the geographical and demographic characteristics that were associated with these patterns.

## Methods

### Data sources

Data consisting of all hospitalizations among children <2 years old in Connecticut from 1997 to 2013 were obtained from the Connecticut State Inpatient Database through the Connecticut Department of Public Health (CT-DPH). Variables included in the data were ZIP code of residence, patient age, primary insurance used for hospital admission, ICD-9 defined diagnoses (up to 10 diagnoses per hospital admission), and week, month, and year of hospital admission.

ZIP-code-level data on total population, population <5 years old, and population that is black were obtained through the United States Census Bureau (by ZIP code tabulation area). These data were used to calculate population density for each ZIP code as well as population density for children <5 years old and percent of population that is black.

In total, there were 28,252 hospitalizations among children <2 years old in Connecticut between July 1, 1997 and June 30, 2013. An RSV case was defined as a hospitalization with at least one of the following documented diagnoses in any of the diagnostic fields: 079.6 (respiratory syncytial virus), 466.11 (acute bronchiolitis due to respiratory syncytial virus), or 480.1 (pneumonia due to respiratory syncytial virus). A total of 9,740 RSV hospitalizations between July 1, 1997 and June 30, 2013 were included in the analysis. The study was approved by the Human Investigation Committees at Yale University and the Connecticut DPH. Certain data used in this publication were obtained from the CT-DPH. The authors assume full responsibility for analyses and interpretation of these data.

### County-level estimates of timing and peak incidence

A harmonic regression analysis was first conducted to evaluate how timing of RSV epidemics differed between the eight counties in Connecticut [11]. With harmonic regression, sine and cosine waves with a specified periodicity (e.g. 52 weeks) are fit to the data. The regression coefficients can then be transformed to obtain estimates of the average amplitude and the average temporal shift (phase) in the curves [11]. Using the county-level data, we fit Poisson regression models with sine and cosine terms that had a period of 52 weeks; the data did not show evidence of overdispersion (deviance/degrees of freedom was near 1), so Poisson regression, rather than negative binomial regression, was appropriate. The average peak timing of the annual epidemics and 95 % confidence interval were estimated for each county [11]. Annual incidence was calculated using the mean annual number of RSV hospitalizations and a proportion of the county population <5 years old to estimate the county population <2 years old [(Mean annual cases / (0.4*population <5))*10,000].

### ZIP-code-level estimates of timing and peak incidence

Building on the county-level analyses, we next used a hierarchical model to estimate the peak timing of RSV and the incidence rate ratios (compared to the statewide average incidence) for each ZIP code. This type of model is well-suited for the relatively sparse ZIP code-level data because information is shared among the ZIP codes during estimation of the associations. For ZIP code *i* at time *t* (in weeks): *Y*_*it*_|*λ*_*it*_ ~ Poisson(*λ*_*it*_), where *Y*_*it*_ is the observed number of RSV cases at ZIP code *i* and time *t*, *λ*_*it*_ is the expected number of RSV cases at ZIP code *i* and time *t*; thus: ln(*λ*_*it*_) = ln(population size_*i*_) + *β*_0_ + *β*_1_ sin(2 * *π* * *t*/52) + *β*_2_ cos(2 * *π* * *t*/52) + *δ*_*i*_ + *η*_*i*_ sin(2 * *π* * *t*/52) + *τ*_*i*_ cos(2 * *π* * *t*/52)

The average peak-time of the annual RSV epidemic in each ZIP code was estimated using the 52-week sine and cosine fixed and random effects coefficients [11]. Incidence rate ratios for each ZIP code compared to the statewide average were estimated by exponentiating the random effect intercept (δ_i_).

### Correlates of peak timing at ZIP code level

We explored associations between ZIP-code-level variations in peak-timing or incidence rate ratios and demographic characteristics. To do this, we used a second stage model, where the estimates of peak timing and incidence from the ZIP code-level hierarchical model were used as the outcome variable. The demographic characteristics that were evaluated were population density of children <5 years old and proportion of the population that is black. We calculated the Pearson’s correlation coefficient (*r*) to evaluate univariate associations and used linear regression to fit a multivariable model.

The analyses were carried out using SAS V9.4, Cary, NC. Maps were drawn in SAS using PROC GMAP. Harmonic regression and linear regression models were fit using PROC GENMOD, the hierarchical model was fit using PROC GLIMMIX, and correlation was measured using PROC CORR.

## Results

### Demographic and disease characteristics

Among children <2 years old hospitalized for RSV, annual RSV incidence in children <6 months old (176.26 cases per 10,000, 95 % C.I. 161.14, 191.39) was more than three times the annual incidence among children 6-12 months old and more than nine times the annual incidence among children 1-2 years old (Table 1). The annual incidence of RSV was highest in ZIP codes where the population density was in the highest quintile (103.27 cases per 10,000, 95 % C.I. 95.71, 110.83). The annual incidence in ZIP codes where at least 20 % of the population was black was more than twice that of ZIP codes where <5 % of the population was black (Table 1). Annual RSV incidence in New Haven County (116.30 cases per 10,000, 95 % C.I. 105.84, 126.76) was almost double the incidence in any other county (Table 2).

**Table 1:** Demographics of the study population^a^

	Number	Annual incidence per 10,000 (95 % C.I.)
Age		
< 6 months	6446	176.26 (161.14, 191.39)
6−12 months	1891	51.71 (46.09, 57.32)
13−24 months	1403	19.18 (16.99, 21.37)
Percent Black		
< 5 %	3488	43.38 (39.63, 47.14)
5−10 %	1494	67.00 (60.17, 73.84)
10−20 %	1562	97.78 (88.16, 107.40)
20 % or more	3143	113.83 (104.65, 123.01)
Population Density		
Quintile 1 (lowest)	174	39.83 (32.13, 47.52)
Quintile 2	369	34.70 (27.42, 41.97)
Quintile 3	907	39.00 (34.29, 43.71)
Quintile 4	2267	45.61 (41.60, 49.63)
Quintile 5 (highest)	6023	103.27 (95.71, 110.83)
		Percent
Insurance		
Commercial	4304	44.2 %
Medicaid	5165	53.0 %
Medicare	2	0.02 %
Other Public	95	1.0 %
Uninsured	174	1.8 %

^a^Numbers may not sum to total due to missing data, and percentages may not sum to 100 % due to rounding. For calculations of incidence, the denominator was the population size for the indicated demographic sub-stratum

**Table 2:** Timing and incidence of RSV in Connecticut Counties

	Peak timing (weeks since July, 1), (95 % C.I.)	Incidence (Annual RSV Hospitalizations per 10,000), (95 % C.I.)
Peak Timing		
Fairfield	28.71 (28.46, 28.95)	68.82 (61.94, 75.69)
New Haven	29.09 (28.90, 29.27)	116.30 (105.84, 126.76)
Hartford	29.09 (28.83, 29.35)	60.73 (54.59, 66.87)
Litchfield	30.12 (29.60, 30.64)	61.10 (48.53, 73.68)
New London	30.70 (30.30, 31.10)	65.26 (56.64, 73.87)
Middlesex	31.13(30.47, 31.78)	50.07 (44.04, 56.09)
Windham	31.18 (30.52, 31.84)	64.57 (54.03, 75.10)
Tolland	31.26 (30.33, 32.18)	28.13 (21.27, 34.99)

**Fig. 1:**
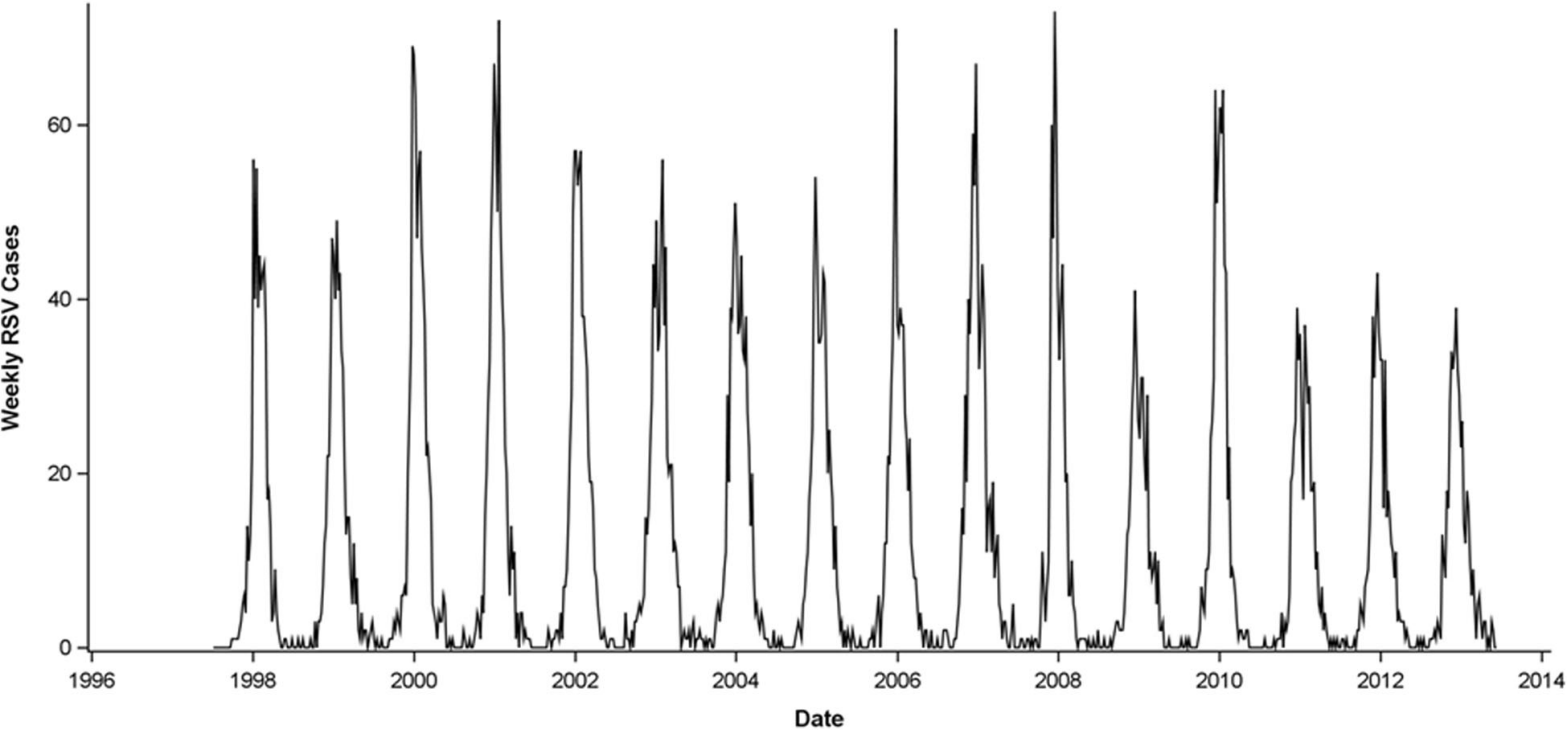
Weekly number of RSV hospitalizations from July 1, 1997 to June 30, 2013

Statewide, the number of RSV hospitalizations per week varied from 0 to 73 (Fig. 1). Seasonal RSV epidemics peaked in late December/early January, and the amplitude of the yearly peak varied by year. The number of RSV cases in a July - June season ranged from 461 hospitalizations in the 2008–2009 season to 764 hospitalizations in the 2006–2007 RSV season.

### Variation in epidemic timing by county

**Fig. 2:**
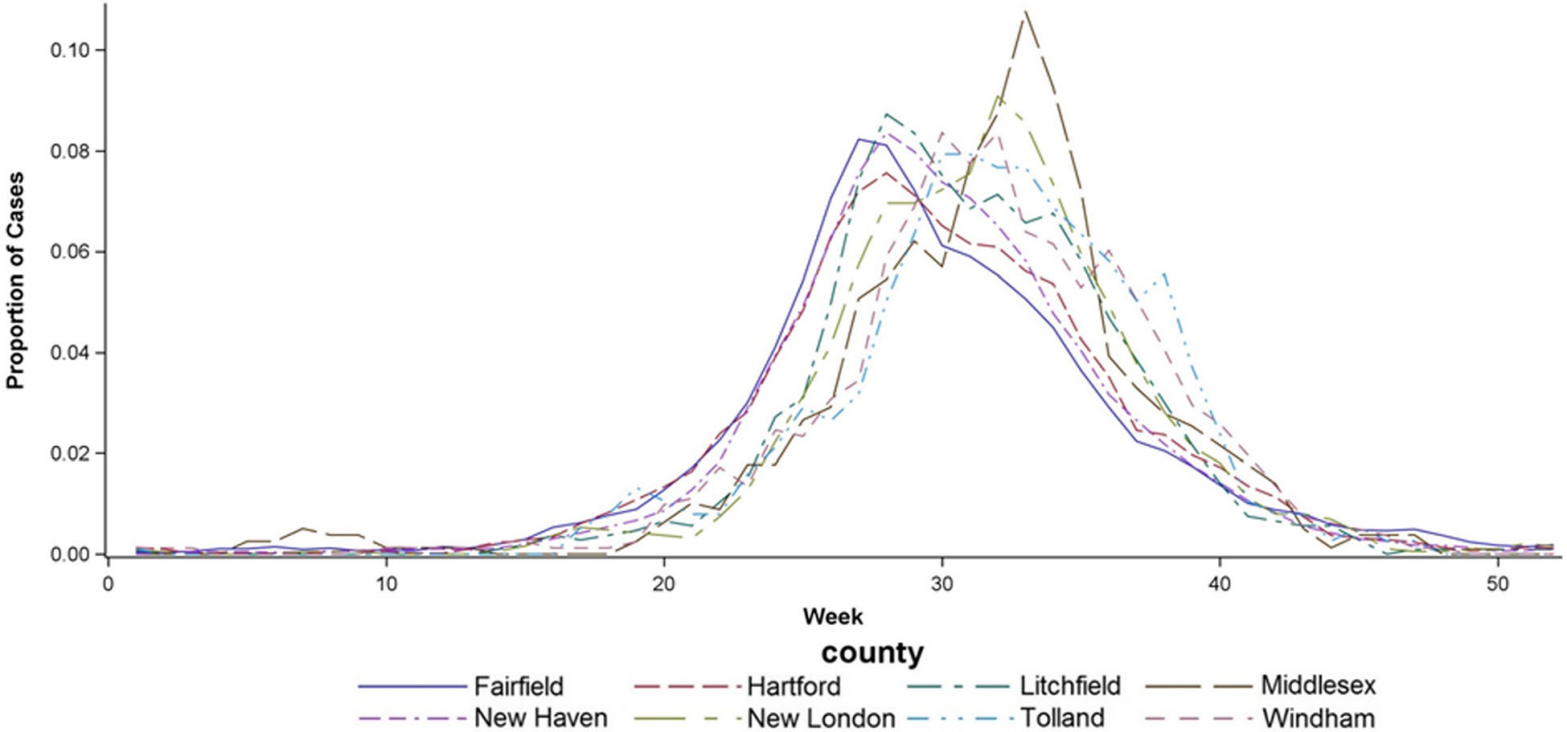
Timing of seasonal RSV epidemics in each of the eight counties in Connecticut. The y-axis represents the proportion of the cases in each county that occurred in each week

The seasonal RSV epidemics peaked earliest in Fairfield county (28.71 weeks after July 1, 95 % C.I. 28.46, 28.95 weeks), 2.55 weeks earlier than the seasonal RSV peak in Tolland County (31.26 weeks after July 1, 95 % C.I. 30.33, 32.18 weeks) (Table 2, Fig. 2). The three counties with the largest populations (Fairfield, Hartford, and New Haven Counties) all had seasonal RSV epidemics that peaked earlier than the other five smaller counties (Litchfield, New London, Windham, Tolland, and Middlesex Counties). Two of the three counties with the earliest peak-timing also had the highest RSV incidence (New Haven and Fairfield Counties).

### Variation in epidemic timing and incidence by ZIP code

**Fig. 3:**
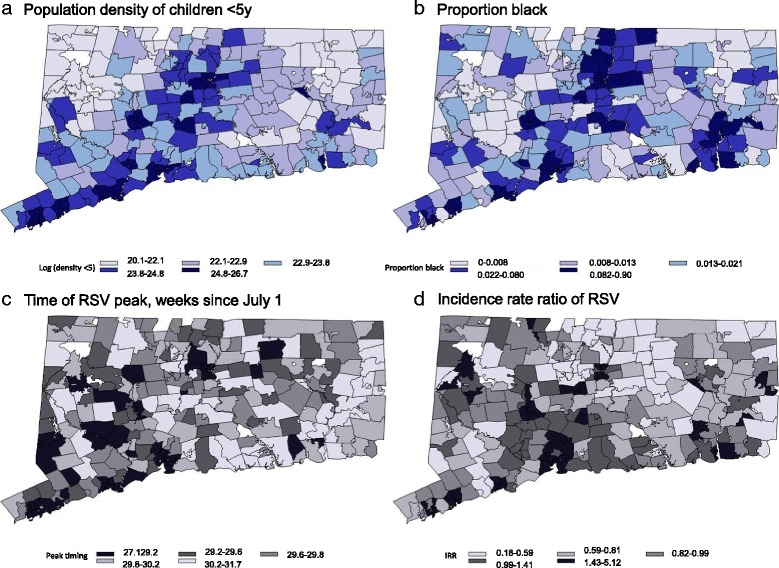
ZIP code-level demographic characteristics and epidemic timing and intensity. **a** log(population density of <5 year old children (**b**) Proportion of the population that is black (**c**) Peak-Timing (weeks) (**d**) Rate ratio compared to the state-wide average

Across all ZIP codes, the average seasonal RSV peak occurred 29.68 weeks after July 1 (Fig. 3). The seasonal peaks among ZIP codes ranged from 27.07 weeks after July 1 to 31.71 weeks after July 1. Large differences in RSV incidence between ZIP codes were also present, as measured by an incidence rate ratio comparing incidence in each ZIP code to the average incidence for the state of Connecticut. The incidence rate ratio varied from 0.18 to 5.18, reflecting rates that were approximately 5-fold above or below average (Fig. 3c, d). Seasonal peak timing and incidence rate ratio were significantly and negatively correlated with one another (earlier peak timing in ZIP codes with higher incidence; r = -0.35, 95 % C.I. -0.46, -0.24).

### Correlates of ZIP-code-level timing

Seasonal RSV epidemics that peaked earlier were associated with higher population density <5 years old (r = -0.45, 95 % C.I. -0.54, -0.34) and higher proportion of the population that is black (r = -0.46, 95 % C.I. -0.55, -0.36). A similar relationship was seen with incidence rate ratios. ZIP codes with higher RSV incidence were associated with higher population density <5 years old (r = 0.46, 95 % C.I. 0.46, 0.55) and proportionally larger black populations (r = 0.58, 95 % C.I. 0.49, 0.65).

Finally, a multivariable model was used to evaluate these relationships. Population density <5 years old and proportion black were independently associated with peak timing. A 1-log increase in population density <5 years old was associated with a peak timing of 0.11 weeks earlier (95 % C.I. -0.18, -0.04 weeks); a 1-log increase in proportion black was associated with the seasonal RSV epidemic peaking 0.13 weeks earlier (95 % C.I. -0.20, -0.06).

Density of children <5 years old and proportion black were also significant predictors of ZIP-code incidence. A 1-log increase in density of children <5 years old was associated with an increase in the rate of RSV of 7.2 % (95 % C.I. 1.0 %−12.7 %). Independently, a 1-log increase in proportion black was associated with an increase in the rate of RSV of 19.7% (12.7 %–25.9 %).

## Discussion

Using a comprehensive statewide hospitalization database from Connecticut, we detected significant local variations in the timing of seasonal RSV epidemics, with more than 4.5 weeks between epidemic peaks in the earliest and latest ZIP codes. The ZIP codes where RSV epidemics peaked earlier tended to be more urban locations with higher population density and proportionally larger black populations. These variations should be taken into account to ensure that prophylaxis for children at-risk of a severe RSV infection is appropriately timed with the local epidemiology of RSV.

Other studies have examined RSV epidemics and individual-level factors that are related with higher RSV risk; risk factors associated with RSV have included sharing bedrooms and having more than 4 children in a house [12], but these are individual-level risk factors that could not be analyzed with ecological data. These measures of crowding could have a similar influence on RSV incidence as population density and population density of children <5 years old. The basic reproductive number (R_0_, a measure of transmissibility) of RSV has also been shown to be significantly associated with population density [13], which could help explain the finding of seasonal RSV epidemics happening earlier in urban areas. Population density and percent of the population that was black are correlates of urbanicity and socioeconomic status of the population in Connecticut. These are likely also correlates of other characteristics of the population that affect the intensity of transmission. For instance, more frequent contacts in dense urban areas or larger household sizes could lead to increased transmission and more rapid epidemic growth.

In order to appropriately provide RSV prophylaxis, it is important to understand when the local RSV season begins as well as how long the season lasts. Our findings on the ZIP-code level in Connecticut build on previous findings on the county level across the US which showed that an earlier optimal time to begin RSV prophylaxis that was associated with urbanization, percent black, and higher population density [10]. Drastic differences in demographic characteristics can exist within counties, so understanding epidemic timing at the ZIP-code level can better guide the optimal time to begin RSV prophylaxis. This type of information can be used to proactively update the recommendations given to providers in advance of the RSV season, allowing for scheduled visits that are optimally timed based on the timing of risk for an individual child. Information on epidemic timing could also be used to determine whether reducing from a 5-dose to a 4-dose regimen would provide adequate protection during the RSV season. In a previous study using national data, we found that a 4-dose schedule would provide protection similar to a 5-dose schedule provided the 4 doses are timed to the local RSV season, and would provide substantial cost savings.

Statistical analyses such as those presented here can be useful to describe variations in epidemic timing and to generate hypotheses. For instance, we have identified an association between population density and the percent of the population in a locality that was black and epidemic timing. These variables are likely correlates of greater urbanization and household crowding and lower socioeconomic status. To better understand the mechanistic drivers of these patterns, models that explicitly capture transmission and host immunity are needed. Such models could be used to test the potential role for specific characteristics (e.g. contact rate, host susceptibility, mixing between adjacent regions) in explaining the observed local variation in RSV epidemic timing.

In addition to ZIP-code level differences in RSV timing, high variability in the duration of RSV seasons at the ZIP-code level has also been observed [9]. Longer RSV seasons have been associated with urban areas, crowding, and percentage of children <5 years old. Analogous mechanisms associated with urban environments and high population density (increased contact with a higher number of individuals, situations in which people live closer to each other) could help explain the similarities observed in RSV epidemic timing and epidemic duration. Insight into the relationship between RSV epidemic timing and duration could provide even better guidance into the optimal use of RSV prophylaxis.

This analysis has both strengths and weaknesses. Through the use of data from the Connecticut State Inpatient Database, this study captured 100 % of hospitalizations in Connecticut. This allowed for the analysis of all hospitalizations of children <2 years old during this time period. Additionally, a large amount of data was available to conduct the analysis. Nearly 10,000 RSV hospitalizations throughout 16 seasonal epidemics provided a large data pool to analyze the timing of, and factors associated with, RSV epidemics throughout the state of Connecticut at the county and ZIP-code level.

There are also weaknesses with the data used and analysis conducted. Since hospitalization data was used, we were reliant on ICD-9 coding. This may lead to some misclassification of RSV cases, as data on viral tests were not available for analysis. Without laboratory testing, other respiratory illnesses such as influenza could have been incorrectly coded as RSV. Furthermore, RSV cases may not have been correctly coded as such and therefore not included in the analysis. Other studies, however, have found good concordance between ICD-9-coded RSV hospitalizations and laboratory data [13]. Additionally, this analysis only evaluated incidence and peak-timing of seasonal RSV epidemics. Since children who are at high-risk of a severe RSV infection need to be protected throughout the duration of seasonal RSV epidemics, understanding the duration of seasonal epidemics is also important to effectively protect high-risk children. Finally, observed differences in RSV incidence could be a result of differences in testing practices in Connecticut hospitals. Hospitals that run broad respiratory panels on all admissions (e.g. Yale New Haven Hospital) might diagnose more RSV, which may help explain differences in reported rates.

## Conclusions

Understanding when seasonal RSV epidemics happen is critical to providing adequate prophylaxis to children who are at high-risk of a severe RSV infection. This analysis can be used to guide policy decisions for when to provide prophylaxis to children who live in different parts of Connecticut. With large differences in peak-timing of the RSV season existing within the state, understanding local RSV patterns (at the ZIP-code level) can allow for prophylaxis to be provided at appropriate times to best protect high-risk children. Further studies that investigate how local RSV timing differs in ZIP codes throughout the United States should be used to appropriately provide prophylaxis based on local RSV epidemiology. By better understanding the factors that predict seasonal RSV epidemic patterns, better, more cost-effective protection can be provided to children who need it.

## Additional file

Additional file 1: SAS code for the hierarchical model. (DOCX 11 kb)

## Abbreviation

RSV: Respiratory syncytial virus
