# Supplementary material for: Local variations in the timing of RSV epidemics
Source: BMC Infect Dis. 2016 Nov 11;16:674. doi: 10.1186/s12879-016-2004-2 (PMC5106786; doi:10.1186/s12879-016-2004-2)
Supplement: Additional file 1: — SAS code for the hierarchical model. (DOCX 11 kb) [file 12879_2016_2004_MOESM1_ESM.docx]

SUPPLEMENTAL INFORMATION: SAS code for the hierarchical model

proc glimmix data = ds1 method = laplace;

class patzip;

model weekcase = sin52 cos52 / dist = poisson link = log offset = log5 s;

random intercept sin52 cos52 / subject = patzip s;

output out = modelzippop pred = predzippop;

ods output parameterestimates = fixedeffectspop solutionr = randomeffectspop;

run; quit;
